# Artificial Interactionism: Avoiding Isolating Perception From Cognition in AI

**DOI:** 10.3389/frai.2022.806041

**Published:** 2022-02-02

**Authors:** Mathieu Guillermin, Olivier Georgeon

**Affiliations:** ^1^Sciences and Humanities Confluence Research Center (EA 1598), Lyon Catholic University, Lyon, France; ^2^UMR5205 Laboratoire d'Informatique en Image et Systèmes d'Information (LIRIS), Villeurbanne, France

**Keywords:** cognitive architecture, intrinsic motivation, constructivism, active perception, constitutive autonomy

## Abstract

We discuss the influence upon the fields of robotics and AI of the manner one conceives the relationships between artificial agents' perception, cognition, and action. We shed some light upon a widespread paradigm we call the *isolated perception paradigm* that addresses perception as isolated from cognition and action. By mobilizing the resources of philosophy (phenomenology and epistemology) and cognitive sciences, and by drawing on recent approaches in AI, we explore what it could mean for robotics and AI to take distance from the isolated perception paradigm. We argue that such a renouncement opens interesting ways to explore the possibilities for designing artificial agents with intrinsic motivations and constitutive autonomy. We then propose Artificial Interactionism, our approach that escapes the isolated perception paradigm by drawing on the inversion of the interaction cycle. When the interaction cycle is inverted, input data are not percepts directly received from the environment, but outcomes of control loops. Perception is not received from sensors in isolation from cognition but is actively constructed by the cognitive architecture through interaction. We give an example implementation of artificial interactionism that demonstrates basic intrinsically motivated learning behavior in a dynamic simulated environment.

## Introduction

From a superficial phenomenological point of view, the subjective experience we have as humans tells us that perception, cognition, and action are three separate realms. Perceiving amounts to merely receiving information about the state of the world. Cognition, then, is about processing this newly acquired information in light of already possessed information, possibly to select an action suited to the current state of the world and to given goals. The appeal of this commonsensical view about perception as isolated and independent from cognition and possible consecutive actions may not come as a surprise. It relates to what Husserl calls the “natural attitude” of day-to-day life, according to which we do not question the existence of objects presented to us in perceptual experience (Husserl, 1982). This natural attitude is irreducible and drives our attention away from any consideration of processes possibly at play in the constitution of experience. Basic biological knowledge reinforces this naïve commonsense picture: sensory organs collect information about the world (perception), that is transmitted to the brain, which processes it (cognition) and drives motor organs (action).

This commonsense model of perception constitutes the ground for an extremely widespread analogy when describing the functioning of a robot or an AI program. It is very common to take a robot's input data coming from its sensors as its perception. The processing of input percepts then stands for the robot's cognition, possibly leading to setting output data traditionally labeled action. Output data drive the robot's actuators. In resonance with the naïve picture sketched just above, sensors stand for the robot's sensory organs, computation for its cognition, and actuators for its motor organs. Echoing our natural tendency to project our intentional life in artifacts we build (Searle, 1980, p. 419), it then becomes tempting to claim that the robot perceives, thinks, and acts as humans do.

This naïve view about perception and cognition does not only influence the general public, feeding fantasies about AI nature and future developments (according to which we would be on the verge of the emergence of strong AI, artificial systems that would reach and exceed the human level). It also influences researchers and engineers and pervades the fields of computer science, robotics, and AI. One of the most influential textbooks in AI defines the latter as the “study of agents that receive percepts from the environment and perform actions. Each such agent implements a function that maps percept sequences to actions (…)” (Russell et al., 2010, p. vii). Russell and Norvig go on:

An **agent** is anything that can be viewed as perceiving its **environment** through **sensors** and acting upon that environment through **actuators**. (…) A human agent has eyes, ears, and other organs for sensors and hands, legs, vocal tract, and so on for actuators. A robotic agent might have cameras and infrared range finders for sensors and various motors for actuators. A software agent receives keystrokes, file contents, and network packets as sensory inputs and acts on the environment by displaying on the screen, writing files, and sending network packets.

We use the term percept to refer to the agent's perceptual inputs at any given instant. (…) By specifying the agent's choice of action for every possible percept sequence, we have said more or less everything there is to say about the agent” (Russell et al., 2010, p. 34–35).

According to this explanation, the algorithm defining actions an agent will perform in response to every possible sequence of perceptual inputs completely describes this agent. This leaves little room for escaping the idea that perception, cognition, and action constitute three separate realms. The agent's cognition lies in the process of selecting actions in response to specific perceptual inputs. Thereby, perceptions and actions are what they are, independent of cognition so defined^1^.

In the rest of this text, we call this conception of perception as radically separated from cognition and action *the isolated perception paradigm*. Despite its pervasiveness in computer science, robotics, and AI, the isolated perception paradigm has been widely discussed and criticized for instance in philosophy and epistemology as well as from within cognitive sciences themselves. A few approaches in robotics and AI explicitly develop alternatives to this isolated perception paradigm. We will review them and describe our original proposition.

Through this paper, we intend to contribute to the study of what it could mean for AI and robotics to take distance with the isolated perception paradigm. We argue that abandoning this paradigm may lead to original ways of designing artificial agents. We thus propose *Artificial Interactionism* as a possible framework that rejects the paradigm of isolated perception. Before detailing this original framework, we first ground our discussion of the possibility to take distance with isolated perception in the broader scope philosophy (phenomenology and epistemology), which provides interesting insights about the relationships between perception, cognition, and action. We then focus upon the manner these relationships are discussed in cognitive sciences and AI, which leads us to bring to the fore the question of extrinsic vs. intrinsic motivations. We finally expose underlying principles of Artificial Interactionism together with the first implementations and experiments.

## Perception, Cognition, and Action in Philosophy

The topic of perception is widely discussed in philosophy, not only through efforts to explain and account for perceptual experience, but also with the study of the role of perception in knowledge acquisition. Let's first focus on interesting insights from philosophical accounts of perception.

### Philosophical Insights Upon Perception

Among typical issues philosophical studies strive to address, one can mention the challenges of describing what are the contents of perception, of explaining perceptual intentionality—the fact that we have experiences about (what we hold to be) objects of the world and their properties—or of elucidating the difference between veridical and non-veridical perceptual experiences.

The first philosophical stream deserving mentioning is *Empiricism* (Lock, Berkeley, Hume, Reid …). Empiricists describe perception as the experience of “*effects* in one produced by things in the world,” namely *sensations* or *sense-data* (Sarkar and Pfeifer, 2006, p. 545). The perceiver is directly aware of sensations or sense data. A strength of this empiricist approach is to provide a way of differentiating (conceptually and in an externalist fashion) veridical and non-veridical experiences. Veridical experiences are experiences of sensations caused by the right objects in the world while non-veridical ones are not. At first sight, empiricists seem to present perceivers as passively experiencing sense data, thus resonating with the isolated perception paradigm. However, things may be less straightforward. To the extent that effects that things in the world cause in perceivers can mobilize their cognitive processes, such approaches may well-depart from isolated perception.

This being said, many thinkers consider that empiricism fails at providing a satisfying account of perception as it does not explain how experiences of sensations (mental or bodily events) can present the world as being a certain way, can provide access to objects of the world and their properties (access to knowledge of the world and its objects). To the extent that perception is subjective contents, experiences of effects internal to perceiver that are caused by interactions with objects of the world, why should we consider that certain sensations present properties of things of the world (like the redness of a fruit) while other do not (like pain when we hit our foot against a stone)? A way of phrasing this difficulty is to say that sensations or sense-data alone are not sufficient to account for the “intentional content of an experience—how it presents things as being” though this intentional content “is given *immediately* with the experience” and is core to perceptual consciousness (Sarkar and Pfeifer, 2006 p. 546–547). A possible answer (that is in fact as much an elimination of the problem) is *phenomenalism*, according to which “objects and states of affairs are themselves just patterns of organization in subjective states of consciousness” (Sarkar and Pfeifer, 2006). “Physical objects are reducible to subjective sensory experiences, or sense-data” (Blaauw and Pritchard, 2005, p. 114). Thereby, perceptual access to, and knowledge of, physical objects are unproblematic. They are not external objects after all.

This discussion directly leads us to the work of Kant, whose account of perception clearly opposes the isolated perception paradigm. With his famous distinction between things in themselves we never perceive and phenomenal objects, Kant could, at first sight, be seen as defending a form of phenomenalism. But, this would miss most of Kant's *transcendental idealism* and its account of perceptual experience. Although Kant argues that we can only perceive the phenomenal world (the world as it appears to us), he nonetheless indicates that we experience objects and states of affairs and that sensations alone are not enough to account for this intentional dimension of perceptual experience (Kant, 1998). According to Kant's description, perceptual experience involves three faculties of the mind allowing acts of synthesis. An act of synthesis is an “act of putting different representations together with each other and comprehending their manifoldness in one cognition” (p. 210). With the faculty of sensibility, the synthesis of apprehension organizes disparate perceptual inputs (*intuitions*) along with the *a priori* forms of space and time. We are never aware of intuitions independently of their organizations within these *a priori* forms of sensitivity. The faculty of understanding then allows the synthesis of recognition in a concept (with at minimum the Categories of the intellect such as the concepts of number, quality, and modality, together with the concept of the object that is recognized). The faculty of imagination permits the synthesis of reproduction for the mind to make the connection between intuitions and concepts.

This Kantian account of perceptual experience also implies that only phenomenal objects are perceived while things in themselves remain irremediably out of cognitive reach. We only perceive objects resulting from the acts of synthesis of the mind. We will return to this epistemological question below. What is important for now is that, for Kant, perception is irremediably conceptualized and entangled with cognition. “Thoughts without content are empty, intuitions without concepts are blind” (p. 193–194). Kant even describes acts of synthesis of recognition under concepts as judgments of *apperceptions* (p. 236). In sum, Kant grants to the mind and its cognitive processes an irreducible active role in perceptual experience. This does not necessarily mean that perception is entirely conceptualized. It is interesting to make the distinction between the intentional content of an experience as it presents the world being a certain way and the phenomenal character of experience, how it *feels* to have this experience (Blaauw and Pritchard, 2005, p. 52). While it is clear that intentional contents require cognition (acts of synthesis), one can appeal to non-conceptual contents such as qualia to approach the phenomenal dimension.

In a similar vein, Husserl also claims that sensations are not sufficient to account for perceptual experience (Husserl, 1982). In fact, an experience, in its intentional dimension (an experience of a given object), can be veridical or not, even though relying on the same sensations. Husserl's phenomenological analysis put the focus on the *natural attitude* we mentioned earlier. Through this natural attitude, perceivers take the experience of the presence of objects for granted independently of the veridical or non-veridical nature of these experiences. This natural commitment to the existence of perceived objects is an irreducible component of perception. Husserl thus argues that phenomenology of perception must study intentional contents of perceptual experiences independently of any assumption about the existence of perceived objects. Such assumptions must be bracketed (Husserl's famous epoché). The fact that an object really exists in the world or not when a perceiver has a perceptual experience of this object shall not enter into the phenomenological analysis of perception. Husserl calls noema this content of perceptual experience phenomenology shall study. As with Kant, Husserl's noema includes, but does not reduce to, non-intentional and non-conceptual sense impressions (hýle). Husserl indicates that perceiving something as a given object requires the background of an intentional horizon composed of the many expectations one has about this object in terms of other possible (future) experiences. Again, the intentional content of perceptual experience irreducibly mobilizes cognition. Although with less intellectual coloration, similar connections between perception and cognition are brought to the fore by Heidegger and Merleau-Ponty (Dreyfus, 2007). Notably, Merleau-Ponty describes a “feedback loop between the embodied coper and the perceptual world” he calls “the intentional arc.” For him, what we learn in our past and what we see possible in our future is “sedimented” in how things look to us (Dreyfus, 2007, p. 1144–1145). Husserl and Merleau-Ponty, like Kant, do not rely on the isolated perception paradigm. The intentional horizon or the intentional arc at play in perceptual experience clearly involves the knowledge and cognition of perceivers.

Remaining on the side of the phenomenological analysis of perception, one can evoke few other elements at odd with the isolated perception paradigm. First, Husserl and Merleau-Ponty can be understood as arguing that cognition is at play when we perceive ourselves as evolving in mind-independent reality. As long as we remain in the realm of phenomenology and Husserl's epoché, whether we really are in contact with a mind-independent reality is irrelevant (it is rather an epistemological question). Thus, notwithstanding their veridical or non-veridical nature, “some experiences are about things in the world external to the mind in virtue of representing possible interaction between the subject and the thing represented” (Siegel, 2021, Section 5.4). Notably, we can take different perspectives on mind-independent objects, which are then expected to take different looks. As we already evoked, such expectations about mind-independent objects enter what Husserl calls the intentional horizon in a quite conceptual approach. In slight contrast, Merleau-Ponty adopts a more action-oriented position, with mind-independence being connected to “‘readiness' on the part of the subject to move her body relative to an object to get a better view, if she so wished” (Siegel, 2021). In addition, one could as well-mention *indexical contents* of perceptual experiences, “contents that must be specified by the use of indexical expressions, such as ‘over there', ‘to the left', ‘here', ‘in front of/behind me', ‘just a second ago', ‘since a few second ago', and so on” (Siegel, 2021, Section 3.4). These indexical contents seem necessary for experience to present spatially and temporally located events. To the extent they appeal to self-consciousness and situation awareness, these indexical contents would involve cognition and their irreducibility in perceptual experiences would mean opposition to the isolated perception paradigm.

### Perception and Epistemology

Let's now turn to epistemological analyses of the role perception plays in knowledge acquisition and the insights they can provide to discuss the paradigm of isolated perception. In this respect, we can start from the “objectivist conception of science” (Baghramian, 2014) that promotes a universal and neutral scientific method permitting us to reliably infer knowledge about reality from empirical evidence and tools of logic. This conception relies on the famous dichotomy between analytic and synthetic statements, between statements “justifiable by *a priori* reasoning” and statements “justifiable only *a posteriori*” on the ground of empirical observation—“*tertium non datur*” (Uebel, 2014, p. 90–91). Arguably, the isolated perception paradigm is at play here. We reach analytic statements through cognition, with results warranted by the application of the laws of reasoning. By contrast, synthetic statements get their epistemic strength from their ground in neutral observation one can tie to isolated perception. Perceptions are reliable because they provide raw information about the world, information that is independent of what observers think or know.

Insights about the limits of the isolated perception paradigm we collected from philosophical accounts of perception are highly significant with respect to the validity of this objectivist epistemological conception. They resonate with many studies from epistemology and philosophy of science. One of the most famous among these is the work of Thomas Kuhn (1996 [1962]), who defends that scientific investigation relies on sets of methods and concepts or taxonomies (together with other elements forming *paradigms*) that are historically situated. In line with the criticism of isolated perception, Kuhn argues that this influence of paradigms reaches perceptual experience itself that relies on concepts available at a given place and time. In consequence, shifts in paradigms (for instance at the occasion of scientific revolutions) can trigger changes in perceptual experience (at least in its intentional dimension; see Bird, 2000, p. 102–104). Through this view he calls the *world-change thesis* (Kuhn, 1996, chapter 10), Kuhn radically opposes the objectivist conception of science: experimental facts collected through perception cannot be considered as neutral grounds for (scientific) knowledge acquisition.

As introduced with Kant's transcendental idealism, the irreducible role of concepts and cognition in perception leads many authors to claim mind-independent reality either is unknowable or does not even exist. We can only know objects as they appear to us (phenomenal objects) depending on our concepts and cognitive processes. However, such conclusions are not inescapable. For instance, the theory-ladenness of empirical observation does not necessarily mean that empirical evidence cannot contribute to theory choice (Brewer and Lambert, 2001). In this respect, it may be fruitful to distinguish between two types of neutrality that one could require from observational statements (Sober, 2014). One can (1) demand a set of observation statements that is independent of any theory and can be employed to test any theory (absolute theory neutrality) or, more modestly, (2) impose mobilizing observation statements that are independent of the theory to be tested (relative theory neutrality). With this second version, cognitive access to mind-independent reality may remain possible. Moreover, one can wonder whether the world-change thesis applies to any possible notion of world or whether one may be entitled to distinguish between scientific worlds that change with paradigm shifts and an empirical ordinary world that is left untouched during such modifications (Ghins, 2003). But, even in such a more optimistic epistemological framework, the detachment from the isolated perception paradigm remains. It is not the role of concepts and cognition that is at stake. It is rather the degree of stability of different sets of concepts.

With his commonsense realism, Putnam also defends the possibility of genuine cognitive access to mind-independent reality despite the acknowledgment of the irreducible role of concepts and cognition (Putnam, 1999). Drawing on the work of American classical pragmatists such as James or Dewey, Putnam deploys an Aristotelian understanding of concepts (Aristotle's direct realism) to which he subtracts essentialism: “when I think [or apperceive] that something is that way, and when the thing is that way, the ‘way' in question is one and the same” (Putnam, 2002, p. 106). In sum, a correct conception is direct cognitive contact with mind-independent reality and allows direct cognitive contact with it in perception under the form of apperception. Apperceiving something involves this something. Apperceiving is a cognitive ability that functions “‘with long arms,' arms that reach out to the environment” (Putnam, 2012, p. 352). We could reformulate this by saying that Putnam put upside down the discussion of issues induced by abandoning the isolated perception paradigm. There is no question about the way we could perceive and know mind-independent reality *despite* the involvement of cognition in perception. Perception and knowledge of mind-independent reality are possible *thanks to* (correct) cognition.

To conclude this brief review of questions associated with the isolated perception paradigm and its rejection, one can recall the epistemological shift operated in physics during the twentieth century. With the development of modern physics (Einstein's relativity and quantum mechanics), the ideal of an observer looking at the world from the outside (as if she was not herself in the world) became more and more problematic (assuming that one could neglect specificities and actions of observers became less and less tenable). With Einstein's relativity, spatial-temporal distances between events are relative to observers' states of movement and situations in gravitational fields. With quantum mechanics, descriptions of particles include reference to observers' choices and actions (to effective experimental settings and devices experimenters settle to observe these particles). By contrast, classical physics was attempting to provide a picture of the world from the “God's Eye point of view” (Putnam, 1981, p. 49) with no reference to observers. The increasing efficiency of experimental devices progressively unveiled that such an ideal was misled and that neglecting situations and actions of observers could be nothing but (at best) an acceptable provisory approximation. Even in the very controlled and refined scientific practice of empirical observation, looking at the world mobilizes very complex networks of components that include observers' situations, some of their cognitive processes, and actions (choices and judgments outcoming in particular experimental structures).

We now propose to explore the manner these first insights from philosophy resonate within the fields of cognitive sciences and AI.

## Perception, Cognition, and Action in Cognitive Sciences and AI

We begin this section by analyzing in more detail the manner the isolated perception paradigm is at play in cognitive sciences and AI. In a second time, we turn to the discussion of alternatives to this paradigm and of the consequences for AI.

### Isolated Perception

To better understand the isolated perception paradigm and its pervasiveness in cognitive sciences and AI, it is interesting to evoke the “foundation of traditional cognitive science” constituted by “the representational and computational model of cognition” (Newen et al., 2018, p. 5). This model pictures cognition as the syntactic manipulation of mental representations. Somehow, the mind computes over a kind of representational “mental code” (Hutto and Myin, 2018, p. 101). This classical cognitivism takes its roots in philosophical views of thinkers such as Descartes—mental representations are the substrates of the mind's activity—and Hobbes—thinking amounts to operating on representations (Hutto and Myin, 2018, p. 96). Though maybe not inescapably, this traditional conception in cognitive sciences naturally leads to the isolated perception paradigm. “According to the standard cognitivist account, information is supposed to be picked up *via* the senses through multiple channels, encoded and then further processed and integrated in various ways, allowing for its later retrieval” (Hutto and Myin, 2018, p. 100). Perception is thereby separated from cognition. It provides information that becomes available for cognition through their encoding in mental representations.

On the ground of this traditional conception, cognitive sciences and AI mutually influenced themselves. The works of Shannon and Turing settled a bridge between the notion of cognition (as the processing of information in so far as it means something) and the work computers can do (the mechanistic processing of information qua physical object). One can also mention Newell and Simon's physical symbol system hypothesis (Newell and Simon, 1976) according to which cognition amounts to the mechanical processing of physical symbols. Through such bridges, both traditional cognitive sciences and AI came to share the tendency to conceive cognition as syntactic-mechanical processing of representations. This idea is often associated with functionalism, “which claims that cognitive phenomena are fully determined by their functional role and therefore form an autonomous level of analysis” (Newen et al., 2018, p. 5). They could thus be adequately described by algorithms susceptible to be implemented in various information processing devices (biological as well as artificial).

This widespread common ground contributed to instating isolated perception as an influential paradigm in AI. Again, if cognition is about processing information through operations upon representations (possibly to select actions to perform), then perception is easily confined to the isolated role of providing contents to some of these representations. One can easily recognize the face of this isolated perception paradigm when Russell and Norvig themselves explain the important role for AI development of the premises of cognitive psychology in the middle of the twentieth century. They mention Craik who “specified the three key steps of a knowledge-based agent: (1) the stimulus must be translated into an internal representation, (2) the representation is manipulated by cognitive processes to derive new internal representations, and (3) these are in turn retranslated back into action” (Russell et al., 2010, p. 13). Cognition occurs only during the second step, in isolation from perception and action. A more recent and emblematic example is the development of AI algorithms capable of playing multiple games through Deep Reinforcement Learning (Mnih et al., 2013). A convolutional neural network is used to learn a control policy directly from sensory input (raw pixels of the Atari game scene). In this example, perception is isolated from the AI algorithm because a ready-made representation of the state of the game is provided as input to the AI algorithm. Moreover, the reward at the end of the game (win, draw, or lose) is also provided as though the agent could directly sense the world state value.

A clarification is in order here. Isolated perception as we intend it in this paper should not be understood as perception independent from computation or information processing. So defined, isolated perception would be an empty notion. Any sensor comes with a certain dose of information processing (at minimum in the filtering it operates by collecting only some of the information available in its environment). For instance, most cameras embed microcontrollers for low-level signal processing (noise filtering, implementation of communication protocols, etc.). Perception cannot be isolated in virtue of being independent of computation or data processing in general. Rather, and more precisely, there will be isolated perception in an (artificial) agent when the computational structures intervening in perception are independent of the rest of the agent's computational architecture in charge of generating outputs (such as selection of actions to perform). This somehow echoes the discussion of the scope of Kuhn's world-change thesis we evoked in the previous section. We saw in this respect that perceptual experience is irremediably mediated by available concepts. Nevertheless, the most radical form of the world-change thesis occurs when mediating concepts belong to, or depend on, sets of concepts empirical observation is meant to test.

Moreover, the depth of computation mobilized in an agent's perception is irrelevant to assess whether the latter is isolated or not. One can for example mention Marr's account of visual perception (Siegel, 2021, Section 8.1) that involves several layers of computation (from 2D levels of gray to reflectance information, then to isometric sketches, and finally up to 3D representations of objects). Though quite complex, this perceptual computation remains in the scope of the isolated perception paradigm as long as it operates independently of a larger cognitive architecture. The same applies even when powerful algorithms, such as pre-trained neural networks, are integrated into sensors themselves (following the logic of Edge computing). In itself, this is not enough to escape the isolated perception paradigm. We could even have isolated perception in an agent equipped with life-long learning of perceptual module based on reinforcement learning (in case the rest of the cognitive architecture of the agent does not intervene in the reward definition).

Now that the notion of isolated perception is clearer (perceptual processes independent from the rest of an agent's cognitive processes, in particular, those in charge of selecting actions to perform), we can turn to what it could mean for AI to take distance with this paradigm.

### Theoretical Alternatives to Isolated Perception

As classical cognitivism is tightly connected with the isolated perception paradigm, let's have a look at approaches in cognitive sciences that reject this traditional conception. We can start by mentioning the different views Albert Newen, Leon De Bruin, and Shaun Gallagher recently gathered under the umbrella term *4E-cognition* (Newen et al., 2018). This stream departs from classical cognitivism by picturing cognition as Embodied (involving the body beyond the brain), Embedded (depending on extrabodily components), Extended (involving extrabodily components), and-or Enacted (with an active role for the agent in its environment). For instance, Hutto and Myin defend “Radically enactive and embodied accounts of cognition, REC” (Hutto and Myin, 2018, p. 96–97) according to which “cognitive processes are not, for example, conceived of as mechanisms that exist only inside individuals. Instead, they are identified with nothing short of bouts of extensive, embodied activity that takes the form of more or less successful organism-environment couplings.” Similar to what Putnam proposes with his commonsense realism exposed in the previous section, with REC “perceiving is a matter of getting a grip on the world as opposed to representing it.” REC clearly opposes the isolated perception paradigm. REC's cognition extends up to organism-environment couplings instead of operating upon the results of perception.

Ecological approaches pioneered by Gibson (1979) and direct perception theories pertain to this stream. They oppose “conventional theories that suppose (at least tacitly) that nervous systems register impoverished, ambiguous, or otherwise inadequate variables of stimulation” (Michaels and Carello, 1981, p. 157). Gibson's pioneering work is directed against Marr's conception of visual perception as a computational process construing 3D representations on the ground of 2D information captured in the retinal images (Sarkar and Pfeifer, 2006, p. 547–550; Siegel, 2021, Section 8.1). According to ecological approaches, perception involves the perceiver as a whole (not just his brain) who sense “structured energy that invariantly specifies properties of the environment of significance to [her]” (Michaels and Carello, 1981, p. 156). For tenants of this line of thought, perception cannot be seen as isolated. The perceiver is “an active explorer of the environment—one who will make an effort to obtain sufficient information” (Michaels and Carello, 1981, p.157–158). This is in line with the shift operated in quantum physics concerning the status of empirical observation whose active nature is fully acknowledged and accounted for. It also echoes theories of *active perception* according to which the brain should not be seen as passively receiving data from or during perception. On the contrary, perception is better conceived of along the analogy of an internet search, the brain playing the role of a query machine. “An agent is an active perceiver if it knows why it wishes to sense, and then chooses what to perceive, and determines how, when and where to achieve that perception” (Bajcsy et al., 2018, p. 178).

The role of action is also core to constructivist theories, such as Piaget's, which propose to keep perception and action entangled in sensory-motor schemes (Piaget, 1954). According to constructivist epistemology, an agent learns through active interaction with its environment and constructs a dynamic data structure that characterizes its current situation (Riegler, 2002). For the same reasons, an agent does not passively receive percepts from the outside world, but actively constructs perception in his inner subjective realm, in compliance with his sensorimotor experience. This type of conception has an interesting resonance with Kant's transcendental idealism and the active role of synthesis it grants to the mind. In this constructivist stream, we shall also mention O'Regan and Noë's famous theory of perception based on implicit knowledge of regular patterns in sensory-motor schemes (O'Regan and Noë, 2001). This constructivist approach strongly resonates with Husserl and Merleau-Ponty's accounts of perception. In granting an irreducible role in perception for active exploration based on available knowledge, it clearly departs from the isolated perception paradigm. Various studies in AI attempt at implementing these Piagetian and constructivist principles (see for instance: Thórisson, 2012; Miller, 2018). More specifically, Piagetian sensorimotor schemes have inspired a range of computer implementations called *schema mechanisms* (Arkin, 1989; Drescher, 1991; Stojanov et al., 1997; Holmes and Isbell, 2005; Guerin and McKenzie, 2008; Perotto, 2013). These implementations have however been criticized for their insufficient account of the core ideas of constructivist epistemology (Bettoni, 1993), as we will examine in the technical discussion in Section Towards an Interactionist Cognitive Architecture. In line with a different interpretation of constructivist epistemology, other authors have suggested following Wiener's Cybernetics theory (Wiener, 1948) and the idea that an agent's input data constitute feedback from action (Powers, 1973; Oyama, 1985; Pfeifer and Scheier, 1994; Laming, 2001; Georgeon and Cordier, 2014; Friston et al., 2017). These authors thus propose an inversion of the interaction cycle in which output data conceptually precedes input data. We will further develop this idea at the technical level in Section Towards an Interactionist Cognitive Architecture.

Beyond the idea of actively constructing knowledge through interaction, Piaget also theorized that agents construct themselves and autonomously develop their intelligence. This idea has been followed up in recent cognitive science with the concept of constitutive autonomy (e.g., Vernon et al., 2015). Froese and Ziemke (2009) argued that constitutive autonomy is necessary for sense-making, which makes it a desirable feature to achieve in artificial agents. Constitutive autonomy implies that the coupling between the agent's cognitive processes and the environment evolves autonomously, meaning that the way the agent perceives the world should evolve through the agent's development.

### Redefining the Goals and Motivations of Artificial Agents

Theoretical alternatives to isolated perception have implications on the goals and motivations of artificial agents. When we delegate our goals to artificial agents we provide them with “criteria and policies for making decisions” instead of explicitly programming them with “every detail of what [they] should do in various circumstances” we give them motivation (Lieberman, 2020, p. 71–72). On this ground, one can distinguish between extrinsic and intrinsic motivation (Blank et al., 2005; Oudeyer et al., 2007; Lieberman, 2020). “Intrinsic motivation is like listening to music. You do it because you enjoy it for its own sake. Nobody has to force you or pay you to listen to music. Extrinsic motivation is when you are motivated for reasons other than the activity itself—you are paid a salary to work at a job, you get a reward, a gold star, social status, etc. (of course, some situations have both kinds of motivation)” (Lieberman, 2020, p. 73).

The extrinsic approach of agents' goals is extremely widespread in AI as it permits solving problems of interest for human designers (board games, self-driving cars …). Most recent AI applications based on machine learning that produced impressive technological achievements belong to this category (e.g., Vinyals et al., 2019). Interestingly enough, typical issues traditional cognitive sciences study also seem to correspond to extrinsic motivation—like playing chess or Hanoi tower, by contrast with problems 4E-cognition tends to focus on such as perception, action, or emotional interaction (Newen et al., 2018, p. 5). In fact, extrinsic motivation is so widespread that intrinsic motivation has yet mostly been used in reinforcement learning as a means to better fulfill extrinsic goals. For example, Kulkarni et al. (2016) have demonstrated the utility of intrinsic motivation to help an agent to win games.

We believe there is a strong connection between this prominence of extrinsic approaches of agents' goals in AI and the pervasiveness of the isolated perception paradigm. Extrinsic motivation problems request artificial agents to reach or produce specific states of the world through the selection of specific actions. It appears like a straightforward first step to divide agents into separated (computational) modules who will (1) operate on representations (some of them being representations of states of the world) to select adequate actions to reach a goal itself defines in terms of states of the world (cognitive module), and (2) perceive the state of the world the agent is in (perception module) to provide inputs to the cognition module. Maybe the connection becomes clearer when we consider the rejection of the isolated perception paradigm. When an artificial agent's perception involves the rest of its cognitive architecture, the designer loses control over the manner the agent represents its environment. Specifying directly goals the agent should reach in terms of states of the world becomes impossible. This makes designs principles that would reject the isolated perception paradigm highly unsuited for the achievement of extrinsic goals. Although it may not be impossible to specify extrinsic goals indirectly, no doubt it would prove extremely (and uselessly?) difficult. By contrast, we think that the rejection of the isolated perception paradigm may open interesting perspectives to design artificial agents with intrinsic motivations. Defining intrinsic goals does not require controlling the representations an artificial agent possesses of the external world. Rejecting the isolated perception paradigm acts as a kind of warrant that goals provided to artificial agents remain intrinsic. Different forms of intrinsic motivation have been proposed, among which curiosity (Oudeyer et al., 2007), the autotelic principle (Steels, 2004), the principle of data compressibility (Schmidhuber, 2010), predictability-based exploration (Bugur et al., 2021), minimization of uncertainty through minimization of free energy (Friston et al., 2017). We have also proposed the notion of *interactional motivation* that seems to us highly compatible with a renunciation of isolated perception (Georgeon et al., 2012). Intrinsic motivation can be seen more like a drive or a value system than as a goal in the sense that it guides the behavior of the agent in an open-ended fashion.

### Alternative Objectives for Research in AI

Now, one could wonder why we, as human designers, should create intrinsically-motivated artificial agents. What could be our goals when attributing intrinsic goals to artificial agents? The answer to this question is manyfold. Intrinsic motivation may enter the design of an AI capable of autonomous mental development (Oudeyer et al., 2007; Nagai and Asada, 2015). Another goal could be to improve our understanding of natural cognition by trying to implement sensorimotor theories of cognition such as the theory of sensorimotor contingencies (O'Regan and Noë, 2001). In the shorter term, implanting intrinsic motivation in artificial agents may allow designing robots who display increased levels of autonomy and with whom it would be interesting to interact and play. To measure progress in such directions we will need a measure of intelligence that does not involve the capacity to reach a predefined goal. The measure should however reflect higher-level behavior than merely controlling a setpoint as it is done in control theory. Chollet's measure of intelligence could be promising as it involves the capacity of the agent to acquire and reuse information on the flow (Chollet, 2019). We could also use subjective evaluation of the agent's behavior by human observers with questions such as “does the agent exhibit curiosity? Playfulness? Does it appear to pursue its own interests? etc..” This would constitute an adaptation of the Turing test to simple activities performed by robots.

## Artificial Interactionism

In this section, we propose a technical approach we call Artificial Interactionism that rejects the isolated perception paradigm and implements intrinsic motivation. It relates to the frameworks of embodied cognition in that the agent learns through active interaction with its environment and constructs a dynamic data structure that characterizes its current situation (Riegler, 2002). We consider this approach to be constructivist and to contribute to the study of artificial developmental learning because the agent autonomously constructs its knowledge of the world and has some room for cognitive constitutive autonomy. We first detail the proposed cognitive architecture before presenting an example of implementation.

### Toward an Interactionist Cognitive Architecture

Our approach integrates the inversion of the interaction cycle design principle introduced in Section Theoretical Alternatives to Isolated Perception. This contrasts with most cognitive architectures that are designed according to a perception-action cycle in which, conceptually, perception precedes action. The interaction cycle revolving indefinitely, one may question why the order matters. This is because inverting the cycle already means taking distance with the isolated perception paradigm. The cognitive architecture's input data do not constitute percepts representing some features of the environment but merely constitute feedbacks from motor controls. Other authors have implemented computer simulations following the inverted interaction cycle design principle (e.g., Porr et al., 2006; Franchi, 2013; Roesch et al., 2013) but, to our knowledge, we propose the first cognitive architecture based on this principle.

More specifically, our implementation rests upon the notion of *primitive interaction*. A primitive interaction works as a reference to a predefined subprogram that specifies how a control loop can be enacted involving both motor control and expected feedback. An example of primitive interaction is moving a touch sensor during a predefined time while receiving a tactile feedback signal within a certain range. By contrast with most other cognitive architectures, the interactionist cognitive architecture does not manipulate isolated percepts and actions. Primitive interactions are the atomic items. The concepts of output data (written in output registers by the robot's software) and input data (read from input register by the robot's software) remain but they are not managed at the level of the cognitive architecture.

Formally, we define the system as a tuple (*S, I, q, v*). *S* is the set of environment states. *I* is the set of primitive interactions offered by the coupling between the agent and the environment. *q* is a probability distribution such that *q*(*s*_t+1_|*s*_t_, *i*_t_) gives the probability that the environment transitions to state *s*_t+1_ ϵ *S* when the agent chooses interaction *i*_t_ ϵ *I* in state *s*_t_ at step *t*. *v* is a probability distribution such that *v*(*e*_t_|*s*_t_, *i*_t_) gives the probability that the agent receives the input *e*_t_ ϵ *I* after choosing *i*_t_ in state *s*_t_. We call *i*_t_ the *intended interaction* because it represents the interaction that the agent intends to enact at the beginning of step *t*; and *e*_t_ the *enacted interaction* because it represents the interaction that the agent records as having been actually enacted at the end of step *t*. If the enacted interaction equals the intended interaction (*e*_t_ = *i*_t_) then the attempted enaction of *i*_t_ is considered a success, otherwise, it is considered a failure. The series of enacted interaction *e*_0_ to *e*_t_ is the only source of information available to the agent about the environment.

There are three major differences between this formalism and the reinforcement learning formalism as it is typically implemented in a Partially Observable Markov Decision Process (POMDP) (Kaelbling et al., 1998): (a) the cycle does not start from the environment but from the agent; (b) the agent's input and output belong to the same set *I* rather than two different sets (observations *O* and actions *A*); (c) there is no reward defined as a function of the states (to avoid isolated perception).

The agent's policy π(*e*_t_, *K*_t_) → *i*_t+1_ is the function that selects the intended interaction *i*_t+1_ based on the enacted interaction *e*_t_ and the data structure *K*_t_. The agent progressively constructs *K*_t_ from the experience of enacting interactions *e*_0_ to *e*_t_. *K*_t_ includes a representation of long-term knowledge learned over the agent's lifetime and a representation of the short-term situation of the agent. We implement π and the knowledge construction mechanism as a *schema mechanism* related to those introduced in Section Theoretical Alternatives to Isolated Perception. Our implementation, however, differs from Drescher-style schema mechanisms which model Piagetian schemes as 3-tuples (pre-perception, action, post-perception). In our analysis, these implementations fall within the framework of isolated perception by directly including percepts in schemes, and, in so doing, they miss an important aspect of Piaget's constructivist theory.

**Figure 1:**
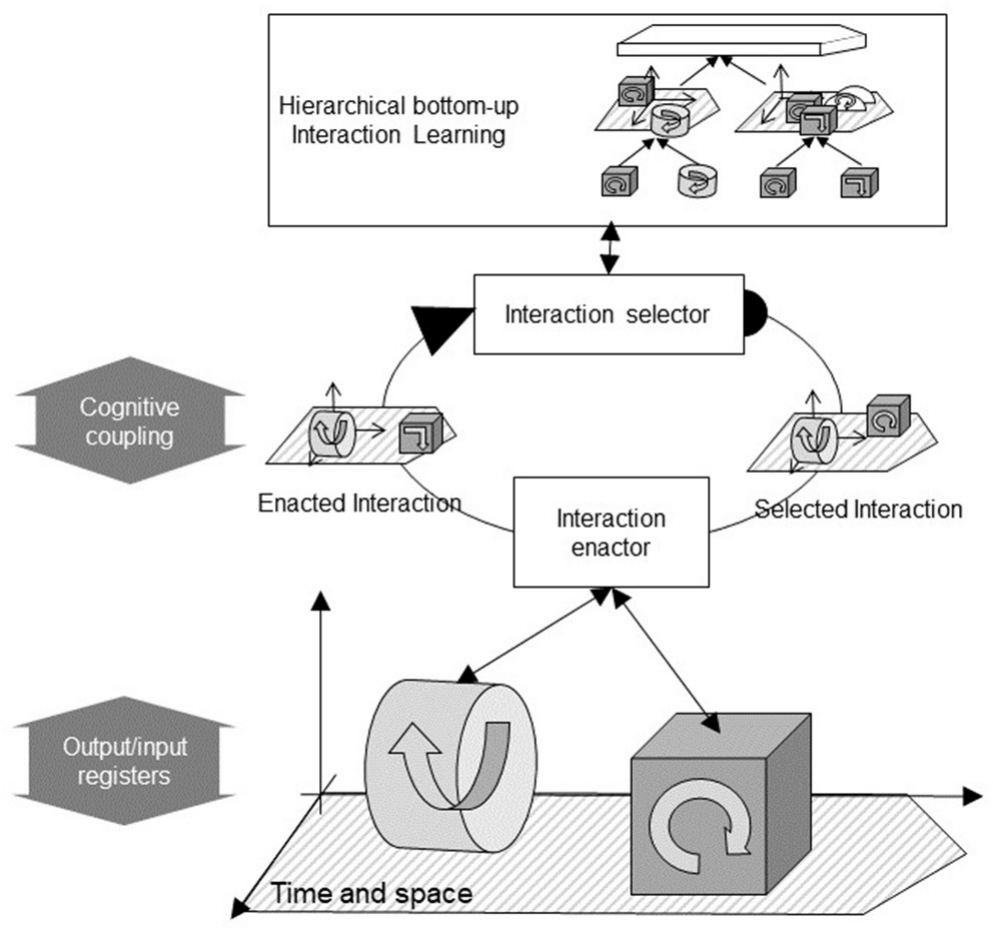
The interaction control mechanism. Cylinders and cubes with arrows represent predefined primitive interactions enacted through control loops. The interaction learning mechanism (top) learns composite interactions (flat arrows) made of hierarchies of interactions organized in time and space. The interaction selector (center) selects a previously learned interaction for enaction (right). The interaction enactor controls the sequential enaction of the selected interaction in time and space (bottom) and returns the enacted interaction (left), which may differ from that selected depending on the environment. The coupling between the interaction selector and the interaction enactor constitutes the cognitive coupling. It is distinct from the software/world coupling defined by the input and output registers.

Instead of 3-tuples, our schema mechanism constructs hierarchies based on 2-tuples (pre-interaction, post-interaction) called *composite interactions*. Composite interactions are recursively recorded on top of primitive interactions in a bottom-up fashion. A first-level composite interaction is a sequence of two enacted primitive interactions (*e*_t−1_, *e*_t_). Higher-level composite interactions are sequences of two lower-level composite interactions all the way up from primitive interactions. We presented the details of this algorithm in a previous paper (Georgeon and Ritter, 2012). This kind of bottom-up pairwise hierarchical chaining has also been studied by Martensen (2020) and Thórisson (2020). Hierarchical composite interactions are illustrated at the top of Figure 1 that pictures our agent's interaction control mechanism.

At any given time, the agent stores a list of recently enacted interactions (primitive and composite) in short-term memory. Interactions in short-term memory are organized in time and possibly in space if information about their approximate location relative to the agent is available. This implies that space and time are amongst the presuppositions hardcoded in the system. In this respect, our approach echoes Kant's transcendental idealism that pictures space and time as indispensable a priori material of perception. In line with Husserl's notion of intentional horizon or with what Gibson called *affordances* (Gibson, 1979), the content of short-term memory constitutes a characterization of the agent's current situation in terms of possibilities of interactions.

Interactions in short-term memory may match the beginning of previously-learned higher-level interactions. When that happens, the continuation of these higher-level interactions is proposed for subsequent enaction. The agent then selects the next interaction to try to enact from among the proposed interactions. The center of Figure 1 illustrates this interaction cycle. The *interaction selector* selects a composite interaction to try to enact from amongst previously-learned composite interactions that are proposed in the current context. The criteria used for selecting interactions define the intrinsic motivation of the agent. For example, if the criteria consist of selecting interactions that have seldom been selected in this context, the robot will exhibit curiosity to try new things; if the criteria are based upon predefined valences of interaction, the robot will appear motivated by hedonist pleasure because it seeks to enact interactions that have a positive valence and avoids interactions that have a negative valence.

The selected interaction is processed by the *interaction enactor* that controls the enaction of the interaction through motor controls sent via the output registers and feedback received through input registers. The bottom of Figure 1 illustrates the enaction of primitive interactions. It is always possible that the attempt to enact an intended interaction fails due to the unexpected configuration of the environment. For example, an interaction consisting of touching an object may fail due to the absence of the object in the expected position. The actually enacted interaction (in this example, moving without touching anything) is returned by the interaction enactor to the interaction selector.

The enaction of primitive interactions takes place at the level of the software/world coupling. The behavior selection mechanism, however, takes place at a higher level called *cognitive coupling*. We define the higher-level policy Π(*E*_T_, *K*_T_) → *I*_T+1_ ϵ *K*_T_ over a higher-level time scale, where *E*_T_ is the previous enacted composite interaction at time *T*, and *I*_T+1_ is the next composite interaction to try to enact. As the agent learns new composite interactions, the cognitive coupling defined by the policy Π rises away from the software/world coupling, as if the agent saw the world in terms of increasingly sophisticated possibilities of interaction.

**Figure 2:**
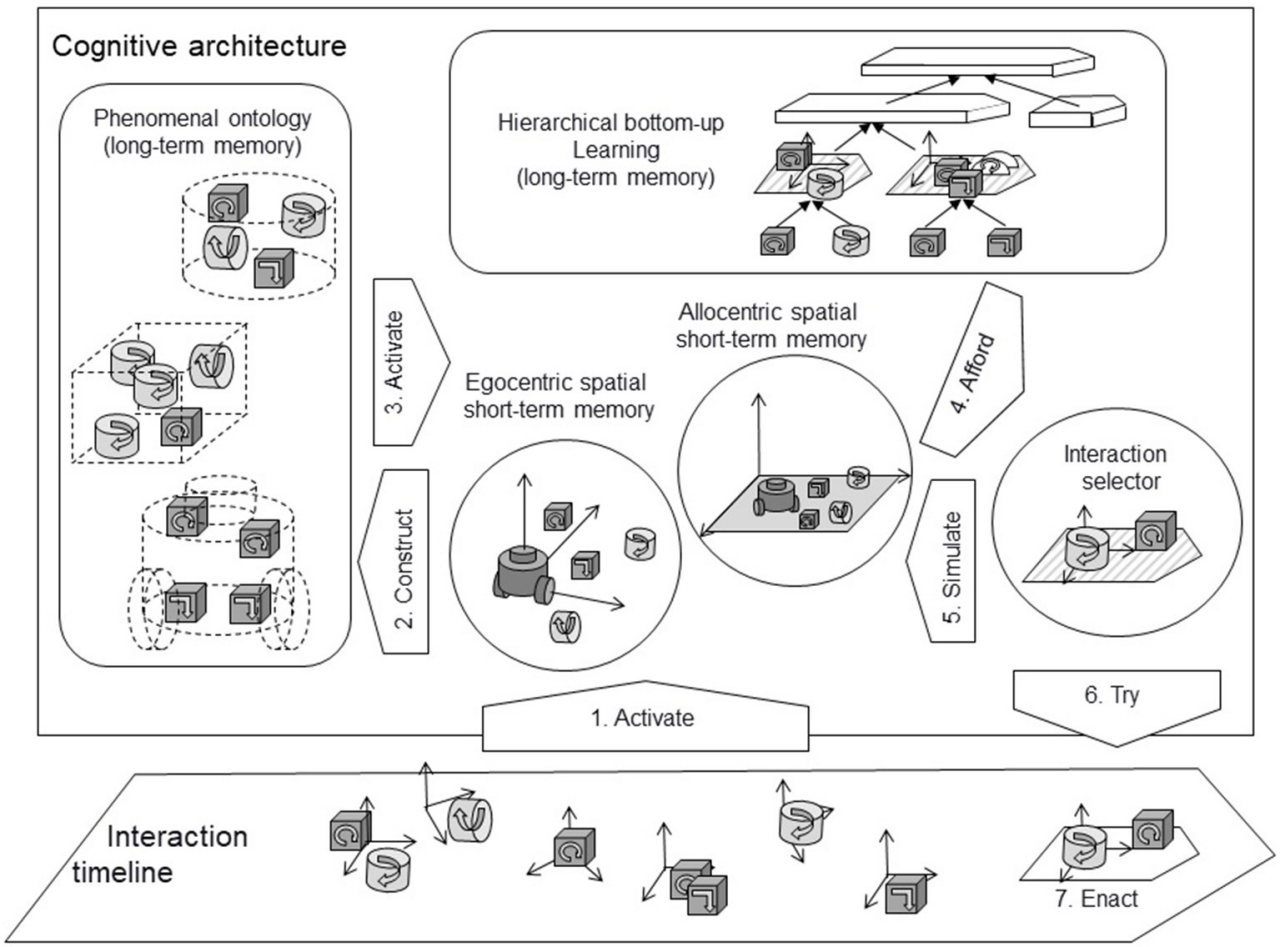
Interactionist cognitive architecture (adapted from Georgeon et al., 2020). As in Figure 1, interactions are represented as 3D blocks along a timeline to highlight the fact that they carry 3D spatial and temporal information. The interaction timeline (bottom) represents interactions as the robot experiences them over time. The cognitive architecture incorporates a hierarchical learning module (top right), different kinds of spatial memories (egocentric and allocentric) used to localize and track the position of interactions (center), and the phenomenal ontology (left) which stores categories of phenomena (objects as they appear through interaction) defined by the interactions that they afford. Enacted interactions activate (1) representations of the context in spatial and temporal memory. Phenomenal representations of objects are progressively constructed (2). When an object is recognized, the interactions that it affords can be activated (3) in spatial memory. The spatial representation of the context affords (4) subsequent composite interactions. In return, afforded interactions can be simulated (5) in spatial memory. The policy Π selects a new interaction, and the robot tries (6) to enact it (7).

From the researcher-designer point of view, an interesting goal is to design agents and robots that have complex shapes and can interact with objects and environments of all kinds of configurations. The robot may need to coordinate different body parts to achieve specific interactions in an environment where different objects may be simultaneously moving. To this end, we built a cognitive architecture to extend the interaction control mechanism presented up to this point in adding specific features suitable to ensure the processing of spatial and temporal information. Like Rudrauf et al.'s (2017) Projective Consciousness Model, our cognitive architecture uses multiple points of view on a spatial representation of the world based on active inference principles. We draw inspiration both from philosophy (notably with the approach of Kant mentioned above) and from biological or cognitive sciences studies. We build upon the analyses of mammalian brains, which evolution has endowed with complex structures to handle space. In return, we expect our models to shed some light on the role those structures play in organizing behavior. For example (Grieves and Jeffery, 2017) list numerous spatially modulated structures in the brain beyond the well-known hippocampus. Such literature suggests that artificial cognitive architecture will need similar functionalities to generate well-adapted behaviors in complex environments. Moreover, authors like Buzsáki and Moser (2013) have emitted the hypothesis “that the neuronal algorithms underlying navigation in real and mental space are fundamentally the same” (p. 130), suggesting that spatiotemporal functionalities could lay the ground toward abstract intelligence. To progress in this direction, we envision the interactionist cognitive architecture depicted in Figure 2.

With this interactionist cognitive architecture, we assume that the robot has some means to locate interactions in space, at least approximately, and to keep track of its own displacements in space (e.g., through an inertial measurement unit as an analogy to the vertebrate's vestibular system). The interaction timeline (Figure 2, bottom) represents events of interaction as the robot experiences them, whether they are initiated by the robot (e.g., moving forward and hitting an obstacle) or not (e.g., the robot is pushed into an obstacle). Information related to the distal sensory system (vision, audition, olfaction) is also handled as interactions that are, as much as possible, located at the source of the interaction—the assumed phenomenon that causes the interaction. This allows the agent to update an internal representation (in terms of interactions) in spatial memory (Figure 2, center), and simultaneously construct temporal hierarchies of sequences of interactions (Figure 2, top). The robot must also construct representations of objects as it experiences them (i.e., phenomena) stored in *phenomenal ontology* (Figure 2, left). Among other types of phenomena, we study how the robot can learn a representation of its own body through interaction and store this representation in phenomenal ontology just as other types of objects. The relations between these different modules are complex and open to research.

In essence, the architecture constructs various data structures that characterize the agent's current situation distributed across several modules: spatial memories, hierarchical temporal memory, phenomenal ontology. These dynamical data structures can be described as a kind of distributed perception that is not obtained in isolation from the other parts of the cognitive architecture. In line with phenomenological approaches of perception of external objects we recalled in Section Perception, Cognition, and Action in Philosophy (notably with Husserl and Merleau-Ponty), this kind of perception also relates to what Endsley (1995) called *situation awareness* in humans, in the sense that these data structures work as operational knowledge of the situation by informing the selection of future behavior based on anticipation of outcomes.

### Example of Implementation

**Figure 3:**
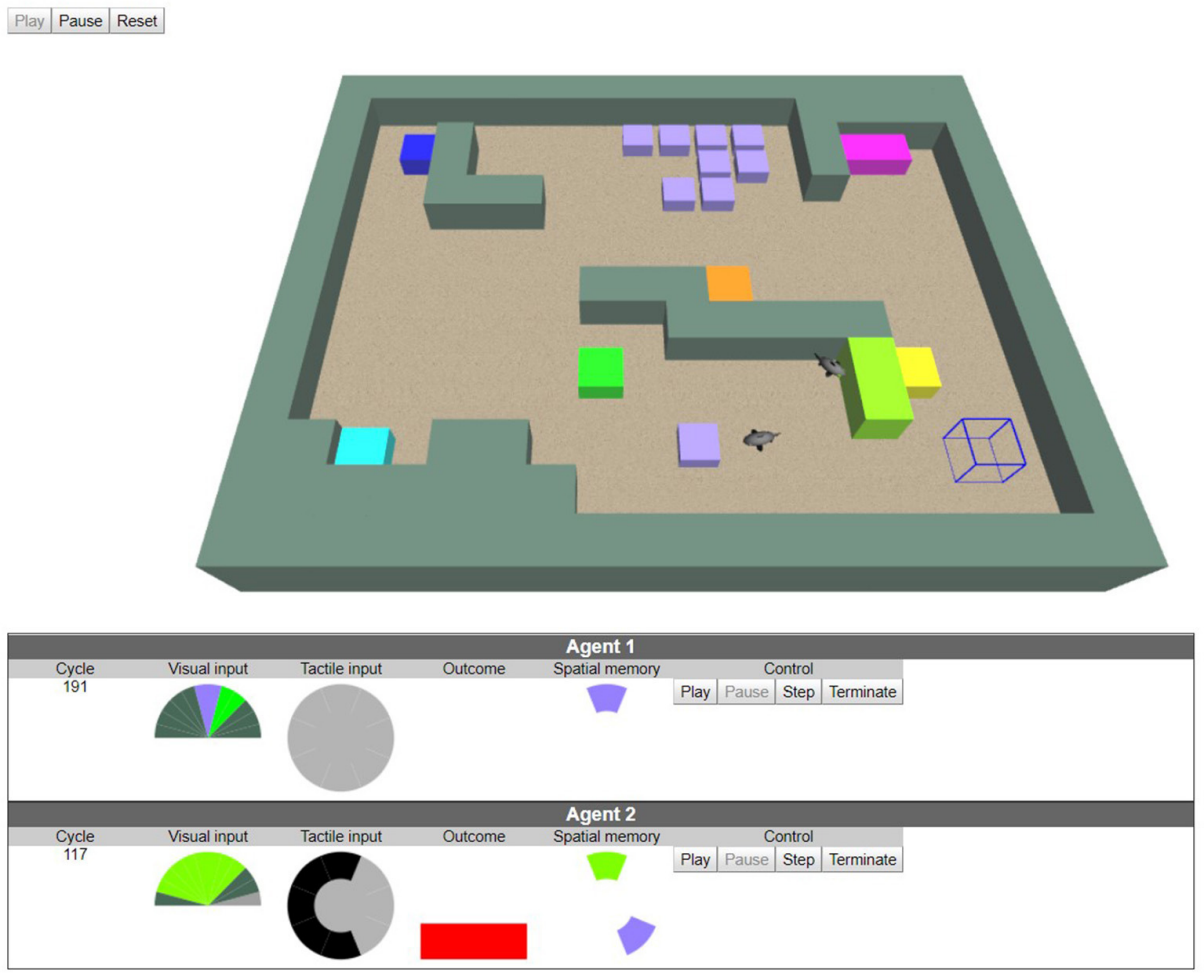
An interactive experiment in a dynamic simulated environment (screenshot from Georgeon, 2021). (Top) two agents (sharks) interact with each other and with three kinds of objects: solid blocks (1 × 1 × 1 cubes), go-through tiles (1 × 1 × 0.5 tiles), food tiles (1 × 1 × 0.5 violet tile). Solid blocks and tiles are opaque to the agent's visual system. The user can add and remove objects and agents in the environment through a mobile cursor (the wired cube). (Bottom) control panels of each agent: visual inputs (12 “pixels” spanning 180°), tactile input (feeling solid blocks around the agent), outcome (bumping into a solid block, socializing by touching another agent, eating food). Egocentric spatial memory (head upward) recording phenomena in the surrounding of the agent. Agent 2's spatial memory shows that Agent 2 remembers the presence of a violet tile at its rear right even though the tile exited the agent's visual field.

We now present an experiment to demonstrate the behavior that can be obtained with the interactionist cognitive architecture. Agents in this experiment figure sharks evolving in a basin, as illustrated in Figure 3. The agent implementation has been described by Gay et al. (2012). It uses the early implementation of the cognitive architecture developed by Georgeon et al. (2013). The experiment was then re-implemented in a 3D dynamic environment originally developed by Voisin (2011) that allows interactions with the experimenter. The reader can interact with this experiment online (Georgeon, 2021).

In simulations such as this one, the control loops that specify primitive interactions can be reduced to a single round of output data followed by input data. In this case, we refer to output data with the term *action*. Although some authors in the cognitive science literature (e.g., Engel et al., 2013) have advocated reserving the term *action* to intentional acts, here we use it to designate mere motor control to comply with the vocabulary used in robotics literature. We refer to the signal received by the cognitive architecture by the term *outcome*. We prefer this term over the term feedback used in cybernetics control theory because the term^2^ feedback is connoted with the purpose of controlling a setpoint, which is not our goal. The outcome may be directly read from the input register or may be computed from input registers by a hard-coded subprogram. Primitive control loops are thus defined by the tuple (action, outcome). Finally, primitive interactions are made of such (action, outcome) tuples associated with spatial information and a scalar valence, as listed in Table 1.

**Table 1:** Actions and outcome.

**Action**	**Outcome**	**Spatial properties**	**Dimensionality**	**Valence**
Impulse forward	Closer	Direction in [–π/2, π/2]	(color index, direction)	Positive
Impulse leftward	Eat	x = 0, y = 0	Boolean	Positive
Impulse rightward	Bump	x = 1, y = 0	Boolean	Negative
	Social	x = 1, y = 0	Boolean	Positive
	Tactile	−1 < x < 1, −1 < y < 1	3^*^3 matrix of boolean	Null
	Default	None		Null

In our experiment, shark agents can perform three actions simulating *fin flaps*: *impulse forward, impulse leftward*, and *impulse rightward*. They however ignore the effects of these actions before experiencing them. The agents' displacements follow a fluid friction law which makes linear and angular speed cumulate over impulses with a decay factor, resulting in a smooth continuous movement. Agents are not tied to the grid but an impulse forward would approximately propel them one unit forward before the next impulse. An impulse sideward would approximately turn them of π/4.

Table 1 summarizes actions and outcomes shark agents can enact. The outcome *bump* is triggered when the agent bumps into a sold block. The outcome *eat* is triggered when the shark enters a violet patch (representing food). The violet patch is then removed. Note that *eating* is not an action but the outcome of the action *impulse forward* when the agent reaches the food as if the agent had a reflex to ingest food. The *social* outcome is triggered when the agent touches another agent with its nose. The *tactile* outcome is a 3 × 3 array of Booleans (representing a somatotopic map) that are set to true by the presence of a solid block in the surrounding of the agent. The *closer* outcome is triggered when a color (different from the background color) enlarges in the visual field.

The valences of outcomes define inborne behavioral preferences one can associate with intrinsic motivations. The positive valence associated with the *closer* outcome gives the agent an incentive to move toward salient objects and thus explore its environment. When the agent reaches an object, other outcomes are triggered, which the agent can then associate together when they overlap in spatial memory. The agent thus performs multimodal integration by learning which interactions are afforded by which objects. The agent learns that solid blocks can be felt through touch, afford bumping, and have specific colors. Violet tiles afford *eating* and *touching* but not bumping. Other agents are gray, afford socialization but not eating nor bumping. Once those associations are recorded (in phenomenal ontology in Figure 2) the agent will tend to move toward objects that afford interactions that have a positive valence (food, other agents), and avoid objects that afford a negative valence (sold blocks).

Gay et al. (2012) reported a detailed analysis of the agent's behavior and learning process. A video with audio explanations can be consulted online (Georgeon, 2014). Note that by defining the valences of interactions, the designer does not specify goal states for the agent, but indirectly defines what goals the agent will tend to reach. Goal-seeking behaviors result from the fact that the *interaction selector* preferentially selects interactions that have the highest positive valence. The user does not directly specify extrinsic goal states. At most, she can lure the agent to specific places by placing *food* in these places.

## Conclusion

In this paper, we have brought to the fore the pervasiveness of what we call the isolated perception paradigm in computer science, robotics, and AI. We studied the possibility, meaning, and consequences of taking distance with this paradigm, drawing on insights from philosophy (phenomenology and epistemology) and recent approaches in cognitive sciences and AI. We notably discussed relationships standing between the rejection of the isolated perception paradigm and the implementation of extrinsic or intrinsic motivations, arguing that this rejection opens an interesting path for designing artificial agents animated by intrinsic motivation. For example, behaviors could be reinforced based on intrinsic-motivation criteria rather than on their efficiency at maximizing an extrinsic reward. Such agents may not be able to win adversarial games but they may fit other purposes such as becoming interesting companions. The community of reinforcement learning could contribute to this endeavor through the rich formalism and techniques that they have developed provided they freed themselves from the tyranny of the extrinsic reward, and imagined other applications than reaching predefined goals. We may learn interesting lessons about cognition in the process.

We proposed a formalism devised from the Markov Decision Process formalism used in reinforcement learning, and a cognitive architecture escaping the scope of isolated perception through the approach we named *artificial interactionism*. Artificial interactionism goes beyond the idea of making perception active and controlled by cognition. It draws on the more fundamental assumption that perception and cognition should not be separated in the first place. This is a more fundamental position that implies revisiting the very fundamental premises of artificial cognitive architectures. Nevertheless, we do not claim that the artificial interactionism paradigm is the only solution to avoid isolated perception. We rather defend it constitutes a proof of concept showing that the separation is not ineluctable. Artificial interactionism offers a general blueprint for designing algorithms to control robots displaying natural behaviors and open-ended learning through interaction. In artificial interactionism, the agent knows its current situation through the enaction of control loops; and has no direct access to ready-made representations of the state of the environment. The lack of access to the environment's state forbids the designer to encode extrinsic goal state recognition criteria. Since the agent is in control of the input data, and not trying to reach a goal state defined within a pre-modeled problem, it is not facing information overload or problem-solving combinatorial explosion. The absence of extrinsic goal states makes room for studying how artificial agents could construct their own goals and also satisfy the objectives of their designers.

Artificial interactionism raises many fascinating questions. An important one bears upon the assessment of the success of intrinsic motivation implementation (possibly by adapting the Turing test and involving human subjective judgments to evaluate the ability of artificial interactionist agents to interact and play with users). More theoretically, one could wonder: should the system be able to learn new primitives, or is it possible to generate well-adaptable intelligence based on a finite predefined set of primitives? How could the agent develop symbolic reasoning and problem solving grounded on interactionist experience? Is it possible to avoid presupposing space in the cognitive architecture (Gay et al., 2017)? In the same vein, an important feature of the artificial interactionism paradigm is that the cognitive coupling can evolve as the agent learns to represent the world in terms of new composite interactions. This opens the question of the possible implementation of cognitive constitutive autonomy (Georgeon and Riegler, 2019).

## Data Availability Statement

The original contributions presented in the study are included in the article/supplementary material, further inquiries can be directed to the corresponding author.

## Author Contributions

OG designed the software implementation and experiment. MG wrote the first draft of the manuscript. MG and OG wrote sections of the manuscript. All authors contributed to manuscript revision, read, and approved the submitted version.

## Funding

MG and OG acknowledge financial support from New Humanism at the time of Neurosciences and Artificial Intelligence project (NHNAI).

## Conflict of Interest

The authors declare that the research was conducted in the absence of any commercial or financial relationships that could be construed as a potential conflict of interest.

## Publisher's Note

All claims expressed in this article are solely those of the authors and do not necessarily represent those of their affiliated organizations, or those of the publisher, the editors and the reviewers. Any product that may be evaluated in this article, or claim that may be made by its manufacturer, is not guaranteed or endorsed by the publisher.

## Footnotes

^1^The quotation from Russell et al. (2010) we reproduced just above comes with a figure that is particularly representative of this conception (therein Figure 2.1, p. 35): it includes the term *percepts* to denote a component impacting sensors (and thus pre-existing the sensors and any form of cognition on the agent's behalf).

^2^The screenshot is from RI Interactive demo. Available online at: http://oliviergeorgeon.com/demo/.
